# Advances in cost-effective integrated spectrometers

**DOI:** 10.1038/s41377-022-00853-1

**Published:** 2022-06-07

**Authors:** Ang Li, Chunhui Yao, Junfei Xia, Huijie Wang, Qixiang Cheng, Richard Penty, Yeshaiahu Fainman, Shilong Pan

**Affiliations:** 1 Key Laboratory of Radar Imaging and Microwave Photonics, Ministry of Education, Nanjing University of Aeronautics and Astronautics, Nanjing, 210016 China; 2 Litin Technology, Xuzhou, Jiangsu China; 3 Department of Engineering, University of Cambridge, 9 JJ Thomson Avenue, Cambridge, CB3 0FA UK; 4 College of Automation Engineering, Nanjing University of Aeronautics and Astronautics, Nanjing, 210016 China; 5 Department of Electrical and Computer Engineering, University of California at San Diego, La Jolla, CA USA

**Keywords:** Integrated optics, Optical sensors

## Abstract

The proliferation of Internet-of-Things has promoted a wide variety of emerging applications that require compact, lightweight, and low-cost optical spectrometers. While substantial progresses have been made in the miniaturization of spectrometers, most of them are with a major focus on the technical side but tend to feature a lower technology readiness level for manufacturability. More importantly, in spite of the advancement in miniaturized spectrometers, their performance and the metrics of real-life applications have seldomly been connected but are highly important. This review paper shows the market trend for chip-scale spectrometers and analyzes the key metrics that are required to adopt miniaturized spectrometers in real-life applications. Recent progress addressing the challenges of miniaturization of spectrometers is summarized, paying a special attention to the CMOS-compatible fabrication platform that shows a clear pathway to massive production. Insights for ways forward are also presented.

## Introduction

Optical spectrometer is one of the most essential instruments in numerous fields, including chemical engineering, materials analysis, astronomical science, medical diagnosis and biological sensing^1–3^. Conventional high-performance spectrometers are typically based on bulky and costly systems with large dispersive components, long optical path length, and movable mechanisms. To meet the requirements of various application scenarios where the portability, cost, robustness, and power consumption are paramount metrics, such as portable or wearable sensing devices for healthcare, food safety monitoring^4,5^, smartphone-based spectrometers, drone-based remote sensing^6^ and space exploration^7,8^, substantial progresses have been made in miniaturizing spectrometers while maintaining adequate performance during the past decades. The projected market for mini- and micro- spectrometers has gone up to about 900 million dollars^9^, which stimulates considerable research efforts from both academia and industry. To name a few, Fraunhofer ENAS announced its goal of batch producing “one gram spectrometer” for smartphone integration at the cost of around one dollar^10^, whilst Rockley Photonics released a whitepaper aiming to realize high-yield manufacturable on-chip spectrometers for “clinic on the wrist” health monitoring^11^. Clearly, efforts are driven toward exploring miniaturized spectrometers that are mass-manufacturable for the consumer market.

In principle, the miniaturization of spectrometers inevitably leads to performance trade-offs in size, operating bandwidth, measuring speed, spectral resolution, dynamic range (or insertion loss), etc. Thus, to achieve commercial products, it is vital to tailor the spectrometer design to suit specific application scenarios. By now, a wide variety of miniaturized spectrometers have been demonstrated, involving diverse architectures, working principles and material platforms, as well as varying technology readiness levels for large-scale manufacturing^12–20^. While some work has indeed advanced the state-of-the-art in miniaturized spectrometers, they may suffer from insufficient performance and low technology readiness levels in connection with commercialization. For instance, ultra-compact spectrometers at the scale of dozens of microns based on single compositionally engineered nanowires have been demonstrated, representing the smallest footprint on record^14^, which for sure, is recognized as a milestone for miniaturized spectrometers. However, a resolution of 5~10 nm is far away from being adequate for applications like glucose monitoring, not to mention gas analysis. Moreover, such technology requires customized epitaxial growth and electron-beam lithography based post-processing, which inevitably results in low yields and high cost at current technology readiness level. Similar concerns also apply to those designs based on colloidal quantum dot^18^ and perovskite quantum dot^19^ given the complexities of their fabrication processes.

To ensure low cost, miniaturized spectrometers must demonstrate a path toward high-volume manufacturing. This generally means lithography-based fabrication and high-level integration. Being compatible with the mature CMOS technologies, silicon photonics provides a promising solution^21^. Besides, the silicon photonics platform features an ultra-high refractive index contrast, facilitating the realization of small device footprints. These advantages can be further extended by introducing other CMOS compatible materials, such as, silicon nitride for its ultra-low propagation loss, broadband transparent spectral window, and insensitivity to temperature variations^22^. Furthermore, by means of heterogeneous integration approaches, III-V gain materials can be heterogeneously or monolithically integrated within the same chip package to implement high-performance light sources and photodetectors^23,24^, enabling densely integrated spectrometer on-chip systems.

Different to other review articles summarizing the general trends on miniaturized spectrometers, this paper pays special attention to the integrated spectrometers that hold great promises for massive fabrication at low cost and specifies their application perspectives. In the following chapter, we will first give a brief market analysis of miniaturized spectrometers and in the chapter after that, application-oriented analysis of miniaturized spectrometers will be provided, including bio-medical monitoring and industrial sensing. In chapter 4, an in-depth technological analysis of miniaturized spectrometers that can be potentially massively manufactured will be performed. The outlook toward developing next-generation integrated spectrometer will be given in chapter 5. Finally, we draw the conclusions in chapter 6.

## Market trends of miniaturized spectrometers

**Fig. 1:**
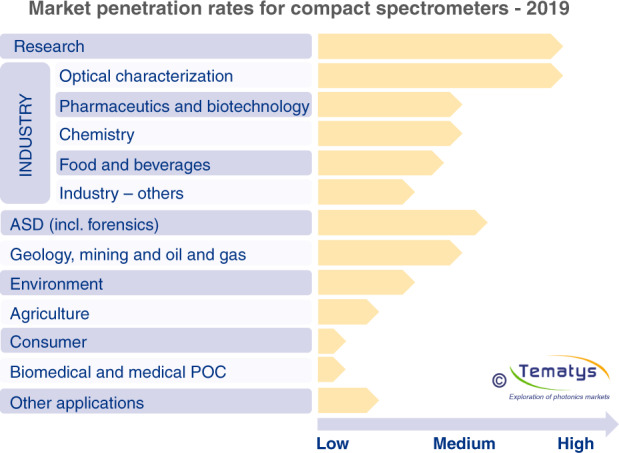
Market penetration rates for compact spectrometers; yellow and red bars represent the rates in 2019 and 2024, respectively. Reprinted from ref. ^9^ with permission from Tematys

Due to the unique light-matter interaction, optical spectroscopy has proven to be an efficient technique to non-invasively analyze compositions of chemical materials, gases, biological tissues, food, etc. The applications of spectrometers were predominantly limited to labs, tests, and metrology, whilst they are now sharply penetrating to consumer market in healthcare monitoring, and food safety. This trend is largely attributed to the development of miniaturized spectrometers, as evident by the rapid growth of the market volume of micro- and nano-spectrometers which arises from 655 million US dollars to 922 million in just three years between 2016 and 2019^9,25,26^. The predicted penetration rates and market values of compact spectrometers in different applications and segments are shown in Fig. 1^9^. The research field has the largest value in 2019 whereas the rapid growth occurs at consumer and biomedical markets almost from 300 to 400% within five years.

**Fig. 2:**
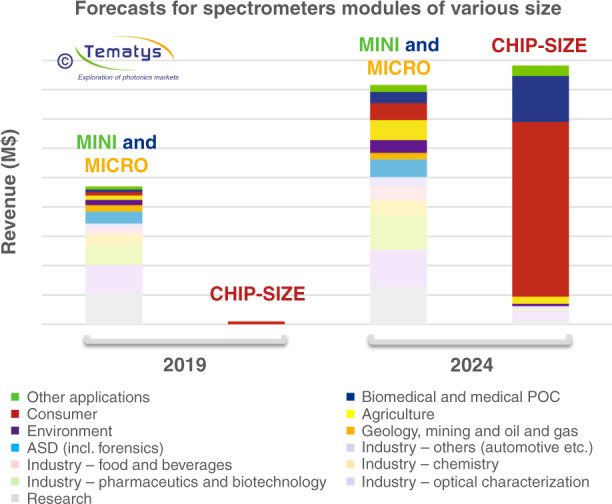
Forecasts for spectrometer modules of various sizes. Reprinted from ref. ^27^ with permission from Tematys

Among those micro- and nano-spectrometers, chip-scale spectrometers are shown to play a dominant role in the rapid market growth in coming years, due to their superior integrability into portable devices such as smartphones, wearable devices and drones, which, on the other hand, is the main driving force behind the development of compact spectrometers. A recent report foresees that chip-scale spectrometers will have a disruptive increase in market volume from less than 2 million US dollars in 2019 to over 1.6 billion US dollars in 2024, as shown in Fig. 2^27^. The rapidly expanding market imposes strong stimulations for both academia and industry players to join this field. Hamamatsu launched a mini spectrometer with a size comparable to fingertip and a weight of only 0.3 g. The device works between 650 nm and 1050 nm with a spectral resolution of about 15 nm, which is suitable for food inspection, light-level measurement and component analysis^28^. NeoSpectra-Micro developed an integrated near infrared spectral sensor (1250–2500 nm) with 16 nm resolution that can be used in a wide variety of material sensing applications for both qualitative and quantitative analysis^29^. Continuous efforts are also being poured into developing low-cost chip-scale spectrometers that can be integrated into consumer electronics such as smartphones. For instance, Fraunhofer-ENAS announced its goal of batch producing “one gram spectrometer” for smartphone integration at a cost of around one dollar^10^. Samsung patented their smartphone-embedded IR spectrometers and prepared to integrate them in Galaxy S11 for skin moisture detection and CO_2_ measurement^27^. In 2020, trinamiX and Viavi Solutions had announced a joint force in building a near infrared spectrometer module targeting for consumer devices. Very recently, Rockley Photonics, a silicon photonics enterprise focusing on telecommunications, released a whitepaper about highly manufacturable on-chip spectrometers for “clinic on the wrist” health monitoring^11^.

All these examples herald the rapid development of chip-scale spectrometers in the future, especially for the consumer and biomedical applications. It is foreseeable that the next-generation integrated spectrometers will be developed on the basis of silicon photonics to enjoy massive productibility at low cost and targeted for the adaptability in ecosystems of intelligent devices such as smartphone and wearable devices

## Application-oriented analysis of integrated spectrometers

Chip-size integrated spectrometers offer great potential for large-scale, in-field, and real-time spectroscopic sensing. Nevertheless, in general, the footprint reduction of a spectrometer consequentially give rise to certain performance degradation regarding its operation bandwidth, resolution, measuring speed, dynamic range, or signal-to-noise ratio, making it crucial to customize the spectrometer design for specified application scenarios. For example, in some cases such as the portable spectroscopies for chemical sensing, a resolution on the scale of 10 nm can be considered acceptable^4^, while for the detection of biomarkers, such as blood glucose or lactate, a sub-nanometer resolution is desired to identify their wide signature absorption peaks (~30 nm) to ensure the measurement accuracy^30,31^. Therefore, it is critical to establish a set of key figures and specify their application-oriented metrics. In this chapter, we discuss the application prospects for integrated spectrometers in different fields, elaborating their key figures of merits in terms of resolution, bandwidth, etc.

By now, the most popular but demanding application scenarios of the integrated optical spectrometers include biomedical sensing for health monitoring and disease diagnosing, chemical sensing for pollution monitoring, gas leak detection and industrial emission screening etc. These detections rely on different vibrational spectroscopies such as near-infrared (NIR, 700–2500 nm), mid-infrared (MIR, 2500–25,000 nm), and Raman spectroscopy (2500–200,000 nm) techniques to measure the relevant markers^30,31^, i.e., to recognize certain chemical substances or functional groups based on the interaction between electromagnetic radiation with vibrational molecules. Among them, since the MIR technique is not suitable for on-chip integration due to the lack of appropriate integration platforms (e.g., the transparency windows for Si or SiN do not cover the broad MIR range), it is not within the scope of this paper. Here we focus on the NIR and Raman spectroscopy techniques that are applicable for chip-size integrated spectrometers and summarize their key spectroscopic performance metrics required for different application scenarios.

### Biomedical sensing

The sensing of biomarkers in either exhaled breath or biofluids, e.g., blood, breath, sweat, saliva, urine, is of great importance for bioanalysis and biomedical diagnosis^32–35^. For instance, the detection of the glucose concentration in blood or saliva can provide diagnostic or monitoring information for diabetes, while measuring the electrolyte level in sweat helps screen for dehydration. These biomedical measurements are commonly achieved by NIR spectroscopy techniques. Since the absorption bands in NIR region are prominently generated by a large number of overtones and combinations of fundamental vibrations of -CH, -NH, and -OH functional groups, the information of various biomarkers can therefore be elucidated accordingly. Benefitting from the maturity of spectrometer integration platform, NIR spectroscopy with portable devices has gradually become a research hotspot, showing promising prospects for in-vivo biomedical sensing.

**Fig. 3:**
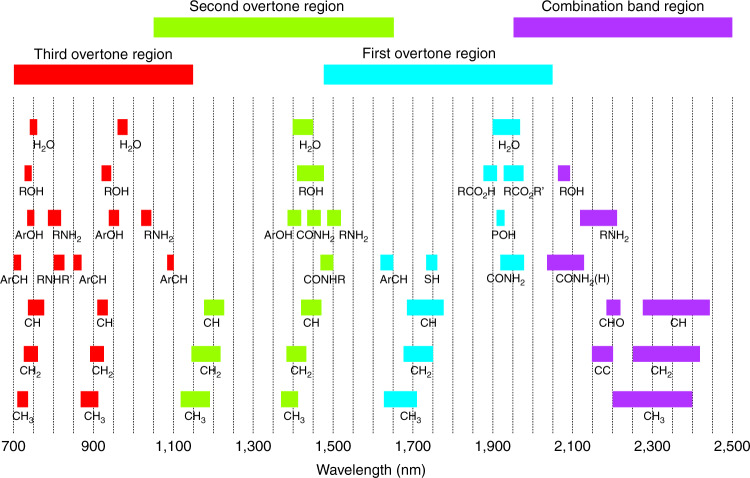
Approximate positions of the vibrational mode assignments of exemplary functional groups in the NIR band. Reprinted from ref. ^35^ with permission from Metrohm

Figure 3 presents the absorption bands for typical functional groups of biological origins in the NIR range^36^. In theory, by identifying the spectral signatures at different bands, quantitative information of corresponded chemical compounds can be retrieved, e.g., the overtones of -CH stretch are associated with carbohydrates while the overtones of -NH stretch relate to proteins. However, given that these peaks in NIR region are rather broad and do overlap, specified spectrometer metrics, as well as additional calibration processes, such as partial least squares regression (PLSR), are needed to extract the mixed bio-information with reasonable accuracy^37,38^. Specifically, such NIR spectrometers should be designed to feature high resolution in particular wavelength regions where the minimum peak overlaps with water or other molecules exist, as well as high sensitivity since the measurable light (either reflected by or transmitted through living tissues) is always rather weak.

**Fig. 4:**
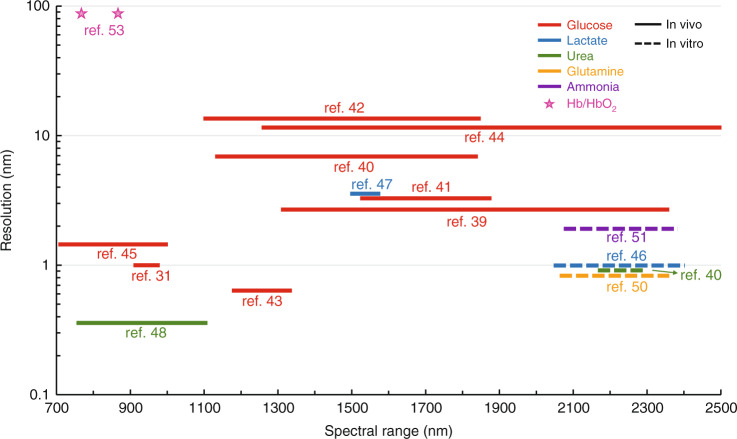
Summary of the spectrometer specifications for the detections of various biomarkers based on published demonstrations. Solid and dashed lines correspond to in-vivo and in-vitro testing, respectively

We first take the measurement of blood glucose as a representative example. As one of the earliest applications of NIR spectroscopy in medical diagnosis, researchers have shown great interests in non-invasive systems for in-vivo blood glucose measurements to alleviate the pain and high cost of conventional invasive methods^38–45^. The informative peaks of the glucose molecule come from the vibrations between -OH and -CH in either long wavelength-NIR (LW-NIR, 1300–2500 nm) or short wavelength-NIR (SW-NIR, 700–1100 nm), representing the two main observe windows on which current analytical methods are based. Relatively, the spectral signatures in LW-NIR are more pronounced than those in SW-NIR which enable higher precision for in-vitro measurement, while the absorption index in SW-NIR bands is notably lower, allowing a deeper penetration depth for in-vivo testing. In Fig. 4, we summarize various reported in-vivo NIR-spectroscopy-based glucose sensing schemes indicating their target spectral bands and required spectrometer resolutions. It can be seen that the SW-NIR based schemes normally require <300 nm bandwidths with sub-nanometer resolutions, while LW-NIR schemes need 300–1000 nm bandwidths with resolutions between 2 and 15 nm.

Besides glucose, NIR spectroscopy has also been demonstrated to extract information about other metabolites, including lactate, urea, glutamine, Ammonia, and (de) oxygenated hemoglobin etc.^46–52^. Since these compounds have lower concentrations in blood or cells compared to glucose, current measurement approaches are mostly based on in-vitro sample testing, which rely on several hundreds of nanometers wide bandwidths with about 1 nm resolutions in the LW-NIR range. Note that in ref. ^53^, the detection of deoxygenated hemoglobin (Hb) oxygenated hemoglobin (HbO_2_) applies only two wavelengths at 730 nm and 850 nm because the absorption spectra at these wavelengths differ significantly between Hb and HbO_2_, and the ratio is then calculated to determine the concentrations. However, this measurement is not accurate on the patients with abnormal hemoglobin structure, abnormal hemoglobin levels.

The above performance requirements can already be met by some commercialized mini spectrometers but are still quite challenging for chip-scale integrated spectrometers. To realize wearable non-invasive devices for in-vivo biomedical sensing, further evolvements in both instrumentation and spectral analysis algorithms are desired.

### Industrial chemical detection

Besides biomedical applications, optical spectroscopic sensing has also been proven as a versatile and powerful tool for high-efficiency industrial chemical detection of various substances. Among them, NIR and Raman spectroscopy are widely adopted and are regarded as a couple of complementary techniques^51^. For instance, the strong infrared absorption of water (H_2_O) always severely interferes the spectroscopic detection and limits the scope of applications whilst Raman spectroscopy is insensitive to water. Therefore, for chip-scale spectrometer, NIR and Raman (800~2500 nm) and their combination spectroscopies are well competent for realizing the high-efficiency chemical detection in a great variety of complicated industrial scenarios^54–59^.

**Fig. 5:**
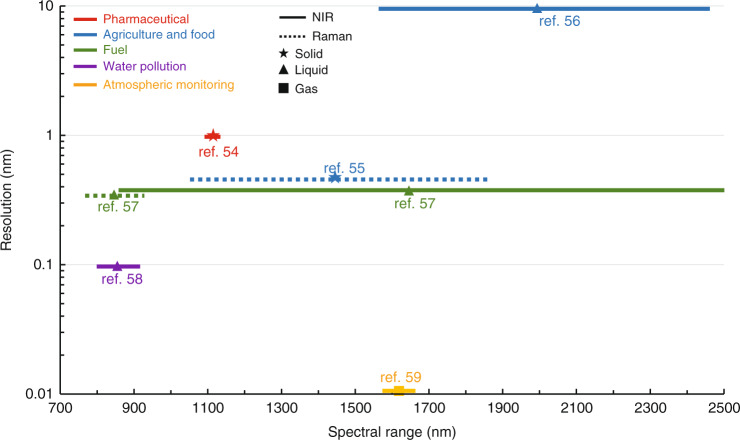
Summary of the spectrometer specifications for industrial applications based on published demonstrations. Solid and dash line represent NIR and Raman spectroscopy, respectively

Figure 5 indicates the spectrometer specifications for industrial chemical detection of solids, liquids and gases based on the NIR and Raman spectroscopy, respectively. The spectroscopic sensing has been utilized to identify and quantify diverse solid, liquid and gas substances for pharmaceutical, agriculture, food, fuel, water pollution and atmospheric monitoring. Most of the scenarios apply spectrometers with a resolution in a range from 0.1 nm to 10 nm. However, the resolution required for gas detection (pm-level) is almost three orders of magnitude higher than that for solids and liquids (nm-level). Since molecules in the gaseous state rotate freely, the fine rotational structures can be observed in the form of fine absorption peaks, especially occurring in the simple molecules (a group of atoms held together by covalent bonds)^60^. So far, the gas detection has been widely demanded by industrial applications, including monitoring the hazardous gases for production safety management, detecting the emission of greenhouse gases and volatile organic compounds (VOCs) for environmental protection^61^. It can be concluded that for industrial applications except agriculture and food analysis, it is always desirable for the spectrometers to have very high resolution (<1 nm) with a bandwidth over 100 nm, which, as mentioned above, remains a great challenge for integrated spectrometers at this moment.

To summarize, the key spectroscopic requirements for both biomedical and industrial chemical sensing applications are listed in Table 1, representing the goals that chip-size integrated spectrometers need to attain. In the following chapters, a variety of integrated spectrometer technologies with diverse operating principles and architectures are discussed, including the state-of-the-art demonstrations and promising development directions.

**Table 1:** Key performance metrics required for different spectral applications

Application scenarios		Bandwidth	Resolution
Biomedical	Glucose	150 nm	14.4 nm
	Lactate	70 nm	3.8 nm
	Urea	125 nm	1 nm
	Glutamine	297 nm	2 nm
	Ammonia	297 nm	2 nm
Industrial chemical	Pharmaceutical	50 nm	1 nm
	Agriculture &food	780 nm	0.45 nm
	Fuel	150 nm	0.25 nm
	Water pollution	130 nm	0.1 nm
	Atmospheric monitoring	90 nm	1.67 pm

## Technological analysis of integrated spectrometers

**Fig. 6:**
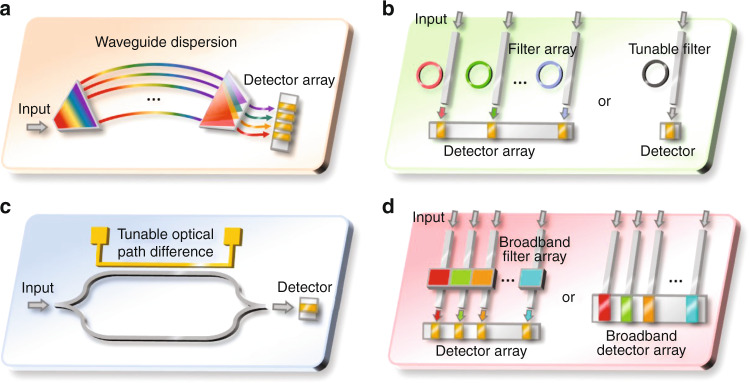
Working principles of on-chip spectrometers. Schematic illustration of the underlying principles for different on-chip integrated spectrometers, including WdM spectrometers based on (**a**) dispersive structure, (**b**) arrayed or tunable narrowband filters, and WM spectrometers based on (**c**) Fourier-transform spectrometers formed by tunable Mach–Zehnder interferometers, and (**d**) broadband filter or detector arrays with distinctive spectral responses for computational spectrum reconstruction, respectively

From the perspective of fundamental technology route, the optical spectrometers can be roughly classified into two groups: wavelength de-multiplexing based (WdM) and wavelength multiplexing (WM) based. As suggested by their names, the WdM spectrometers need to de-multiplex, or in other words, split the incident signals’ spectra, either spatially or temporally, and measure the intensity of individual channel. On-chip WdM spectrometers are typically implemented by dispersive structures, in which different wavelengths exhibit different propagation properties and consequently arrive at detectors distributed at different spatial locations or reach a single detector at different time. Alternatively, WdM spectrometers can be implemented by using an array of narrowband filters or a single tunable narrowband filter, whose spectral responses determine the spectral contents arriving at the detector, as shown in Fig. 6a, b. In contrast, WM spectrometers do not require to split the spectral contents of the spectrum, they typically pro-modulate the entire spectrum and reconstruct it by signal processing using specific algorithms. Dependent on the modulation principle, they can be further divided into Fourier Transform Spectrometers (FTS), or Computational Spectrometers (CS) as shown in Fig. 6c, d. Note that, in the figures, only the core parts of the integrated spectrometers are illustrated, which refer to the integrated optical elements that are required to performance the spectrum reconstruction and are typically unique for different type of spectrometers. While a complete system of integrated spectrometer contains other necessary auxiliary elements or devices, such as optical I/Os which couple light in and out of the chip, photodetectors (either on- of off-chip) that convert light to electrical signal for post-processing, driving electronics, tuning elements and temperature controller.

### WdM spectrometers

Being the most straightforward spectroscopic approach, integrated WdM spectrometers don’t require complex computational sources, thus have attracted numerous research attention to be demonstrated on silicon photonics platform. Till now, gratings are the most widely adopted elements for dispersive structures based WdM spectrometers. The fundamental principle is to create multi-path interference effects between the incident signal and make different wavelengths arrive at different locations. Depending on how the multi-path interference is created, gratings based spectrometers can be further divided into planar concave grating (PCG) and arrayed waveguide grating (AWG).

**Fig. 7:**
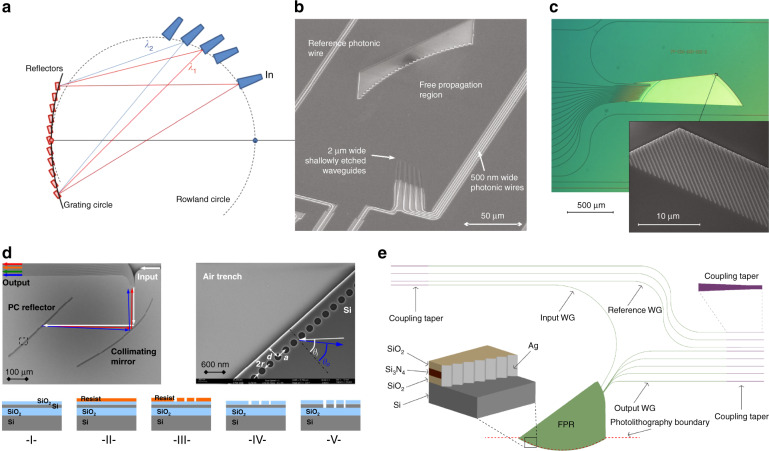
PCG based spectrometers on silicon photonics platform. **a** Schematic illustration of a PCG spectrometer. **b** SEM image of a silicon PCG spectrometer^62^. **c**–**e** PCG spectrometers with Bragg reflectors^63^, photonics crystal reflector^65^, and coating grating with silver films^66^, respectively. **b** Reprinted from ref. ^62^ with permissions from IEEE Publishing. **c** Reprinted with permission from ref. ^63^. Copyright 2014 American Chemical Society. **d** Reprinted from ref. ^65^ with permission from IOP Publishing. **e** Reprinted from ref. ^66^ with permission from IEEE Publishing

PCG is the planar version of the classic diffraction grating spectrometer (such as Optical Spectrum Analyzer used in daily lab). The light enters from an input aperture into the free propagation region (a slab waveguide section) after which it diffracts and hits the curved grating on the other end, which reflects as well as focuses different wavelengths at different output apertures located along a so-called Rowland circle as shown in Fig. 7a, b. The spectral resolution is linearly dependent on the pitch of the output aperture (spacing between two output waveguides) and inversely proportional to the Rowland radius that determines the device footprint as well as the group index of the material. Therefore, demonstrating PCG on silicon photonics can offer significant reduction in footprint thanks to the high field confinement in the waveguide core that leads to a small output pitch and the strong modal dispersion the generates a large group index. The first experimental demonstration on silicon photonics was in 2007 consisting of 4 channels with 20 nm channel spacing (i.e., spectral resolution) and a footprint of only 250 μm × 180 μm^62^. The main research focus since then have been given to improve the spectrometer’s performance by optimizing the gratings, including using Bragg reflectors^63,64^ or photonics crystal reflectors^65^, coating grating with silver films^66^, Retro-reflecting V-shaped facets^67^. etc., as plotted in Fig. 7c–e.

**Fig. 8:**
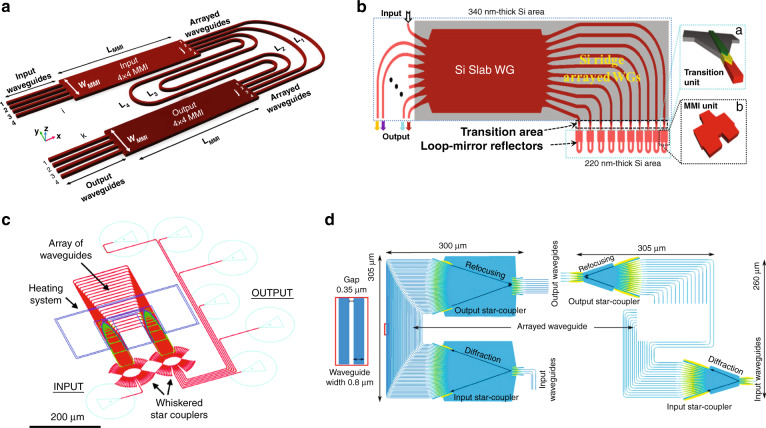
AWG based spectrometers on silicon photonics platform. **a** AWG with MMIs for compact footprint^68^. **b** AWG with reflectors to reduce half of the size^70^. **c**, **d** AWGs with advanced star couplers and wide waveguides to reduce channel cross-talk^71,72^. **a** Adapted from ref. ^68^ with permission from IEEE Publishing. **b** Reprinted from ref. ^70^ with permission from IEEE Publishing. **c** Reprinted from ref. ^71^ with permission from IEEE Publishing. **d** Reprinted from ref. ^72^ with permission from IEEE Publishing

In contrast with PCGs that use a slab waveguide section for the multi-path interference to take place, AWGs use an array of waveguides with varying lengths to create the multi-interference effect as shown in Fig. 6a. Light enters from an input waveguide into a free space region and gets distributed among the imbalanced arrayed waveguides through in-plane diffraction. The phase shifts contributed by the arrayed waveguides are wavelength dependent, consequently, the light coming out of the arrayed waveguides interfere in another free space region, such that different wavelengths arrive at different output apertures. The length difference between the waveguide arms (delay length) relates directly to the Free Spectral Range (FSR) or the operation bandwidth of the AWG, while at a given FSR, the spectral resolution can be simply improved by increasing the number of waveguides. The benefit of using silicon photonics as the platform for AWG lies in the high confinement of light in the waveguide core, allowing tight bend radius and narrow waveguide spacing. In 1997, the first AWG on silicon photonics was demonstrated consisting of 4 channels spaced at 1.9 nm with a footprint of 2.7 cm × 2.7 cm^40^. Compared with PCG, AWG is relatively simpler in design and resolution improvement. However, it has larger footprint as it incorporates two free propagation regions and an array of long waveguides. AWG is also more vulnerable to fabrication variations that induce phase errors along the arrayed waveguides, this is why AWG typically exhibit stronger channel crosstalk than PCG. Various innovations have been created to combat those problems. For instance, the footprint reduction can be achieved by replacing the free propagation regions with multi-mode interferometers (Fig. 8a)^68^ and connecting the ends of the arrayed waveguides to reflectors (Fig. 8b)^69,70^, while the crosstalk can be suppressed by using advanced free propagation regions as well as wider waveguides, as shown in Fig. 8c, d^71,72^. This type of integrated spectrometers using PCG or AWG can perform single-shot measurement as no tunning elements are present, thus the measurement speed can be fast. The system’s daynamic range is directly related with its operation bandwidth and resolution, as for broader bandwidth and higher resolution, the incident light needs to be split into more ports, resulting in lower detected power at the detectors. The main limitation lies in the relatively short propagation path compared with bulky system, which is the key factor toward high resolution, as longer waveguide length will inevitably bring more propagation loss and phase errors due to fabrication imperfections, which in turn sacrifice the system’s dynamic range and reliability. As a consequence, the resolution of this type of integrated spectrometers is typically limited to a few nm.

**Fig. 9:**
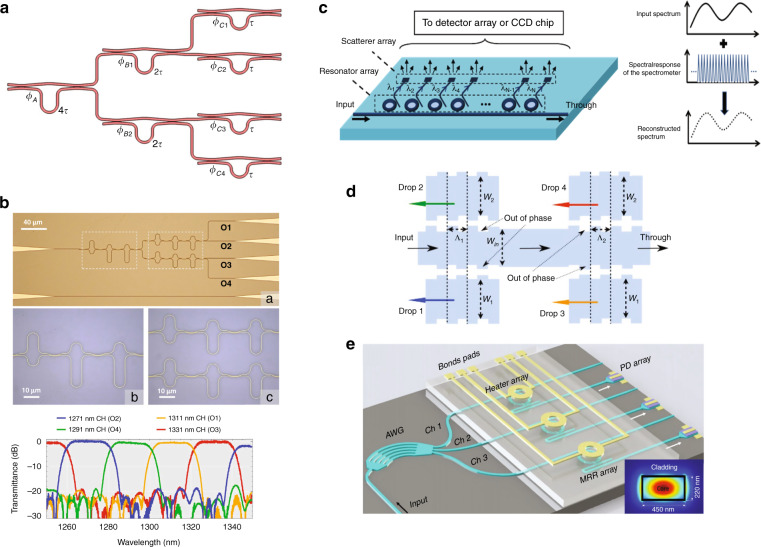
Narrowband-filters based spectrometers on silicon photonics platform. **a** Schematic of a cascaded MZI filters based spectrometer^73^. **b** Spectrum tailoring of cascaded MZI filters based spectrometers by optimizing the couplers at each MZI^74^. **c** Schematic of a ring resonators array based spectrometer^79^. **d** Cascaded Bragg gratings based spectrometer^80^. **e** Spectrometer composed of both AWG and ring resonators^81^. **a** Reprinted from ref. ^73^ with permission from IEEE Publishing. **b** Reprinted from ref. ^74^ with permission from IEEE Publishing. **c** Reprinted from ref. ^79^ with permission from Optica Publishing Group. **d** Reprinted from ref. ^80^ with permission from Optica Publishing Group. **e** Reprinted from ref. ^81^ with permission from IEEE Publishing

Besides dispersive gratings, narrowband filters can also be developed toward WdM spectrometers. The most important advantage of filters based spectrometer over dispersive gratings based spectrometer is the flexibility in tailoring the spectral response. For instance, by cascading Mach–Zehnder-Interferometers (MZIs) with varying armlength differences, spectrometers with spectral resolutions from 0.13 nm to 20 nm can be easily synthesized^73–75^, as shown in Fig. 9a. By further optimizing the couplers in MZI, the spectrum of each channel of the spectrometer can be tailored to flat-top instead of being Gaussian or Lorentzian shaped as those of dispersive gratings based spectrometers^74^, which is plotted in Fig. 9b. MZI is a non-resonant filter which intrinsically occupies large area, while using resonant filters such as ring resonators, Fabry-Perot etalons, photonics crystal cavities and Bragg gratings can significantly reduce the linewidth as well as the footprint^76–78^. Fig. 9c shows a compact spectrometer consisting of 84 micro-donut resonators that could produce 0.6 nm resolution within 50 nm operation bandwidth and only occupies a footprint of 200 μm × 50 μm^79^. 4 cascaded contra-coupled Bragg gratings can also achieve similar spectral responses compared with the spectrometer consisting of over 10 cascaded MZIs but has a footprint that is one order of magnitude smaller^80^, as shown in Fig. 9d. Spectrometers based on narrowband filters array have the advantages of single-shot measurement (fast measurement speed) as well as flexibility to tailor the resolution and bandwidth. Particularly for resonant filters, it can be easily achieved ~10 pm resolution. But at the same time, they are vulnerable to fabrication variation that changes the operation wavelength as well as ambient environment fluctuations that can induce instability of the operation wavelength. Also, they have similar problem with PCG or AWG based spectrometers that the dynamic range is directly related with operation bandwidth and resolution.

Another unique advantage of narrowband filter based spectrometer compared with dispersive gratings lies in the tunability of its operation wavelength, particularly for resonant filters, which makes scanning spectrometer that uses a single filter with ultra-compact footprint and large dynamic range possible. However, for filters with very narrow linewidth (high spectral resolution), tuning the wavelength for a broad optical range will require a significant amount of power consumption and the range will typically be limited by its free spectral range. Therefore, the trend of combining dispersive gratings and narrowband filters for spectrometers with both broad bandwidth, high spectral resolution and small channel count is arising. The dispersive gratings are intended to have low-resolution and split the spectrum into multiple channels with large bandwidth, while a narrowband filter finely scan each entire channel by tuning its center wavelength. Recently, the combination of a AWG and ring resonators was demonstrated for 0.1 nm resolution and 25.4 nm bandwidth with only 9 channels as shown in Fig. 9e, which is significantly reduced compared to a conventional AWG that would require 254 channels^81^.

### WM based spectrometers

The fundamental principle of WdM spectrometers requires the spectrum to be split into multiple channels, which limits the power at each detector and the dynamic range of the spectrometer. In contrast, WM spectrometers pre-process the entire spectrum of the incident signal by certain type of modulation and reconstruct the spectrum using proper algorithms to post-process the modulated spectrum. Based on the specific modulation, WM based spectrometers can be categorized as Fourier transform spectrometers (FTSs) and Computational spectrometers (CSs).

#### Fourier transform spectrometers

**Fig. 10:**
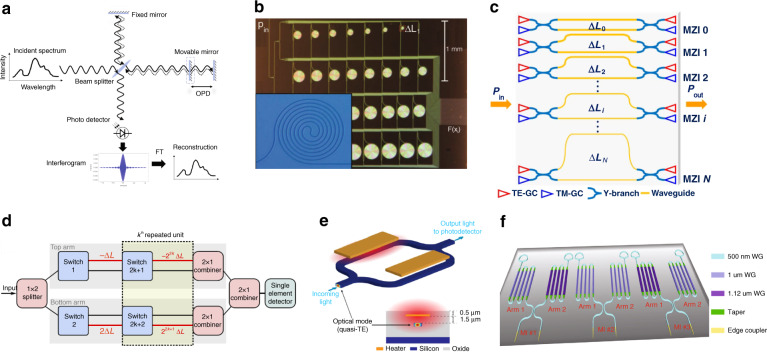
Fourier transform spectrometers on silicon photonics platform. **a** General framework of a FTS^17^. **b**, **c** FTS on silicon photonics with OPDs introduced by an array of imbalanced MZIs^15,83^. **d**, **e** FTS on silicon photonics with OPDs tunable in time domain^16,118^. **f** The state-of-the-art work on silicon TH-FTS with 180 nm bandwidth and 0.16 nm resolution^17^. **a** Reprinted from ref. ^17^ with permission from John Wiley and Sons. **b** Reprinted with permission from ref. ^83^. © Optica Publishing Group. **c** Reprinted with permission from ref. ^15^. © Optica Publishing Group. **d** Reprinted by permission from Springer Nature: Nature Communications ref. ^118^, 2018. **e** Reprinted by permission from Springer Nature: Nature Communications ref. ^16^, 2018. **f** Reprinted from ref. ^17^ with permission from John Wiley and Sons

FTSs modulate the incident light via tuning the optical path difference (OPD) to obtain the interferograms and then convert them back to the wavelength domain by performing Fourier transform in the post-processing stage, as shown in Fig. 10a^82^. Compared with WdM spectrometers that commonly measure different spectral components separately in the temporal or spatial domain, the multiplexing measurement of FTSs can provide much better SNR with higher optical throughput captured by the detectors, especially when the detector noise is dominant, which is identified as the Fellgett’s advantage (multiplex) and the Jacquinot’s advantage (high optical throughput). According to the Rayleigh criterion, the spectral resolution δλ and bandwidth Δλ of FTSs are determined by the maximum OPD introduced to the signal and the number of OPDs, respectively. For conventional free-spaced FTSs, the OPD modulation can be easily performed using a movable mirror as shown in Fig. 10a. While demonstrating FTSs on integrated planar platform such as silicon photonics will require to identify approaches to implement variable OPD without mechanically moving parts. Depending on the mechanism of introducing various OPD to the signal, FTSs demonstrated on silicon photonics can be further categorized into spatial heterodyne FTSs (SH-FTS) and temporal heterodyne FTSs (TH-FTS).

SH-FTS are based on a simple and stable multi-interferometer configuration composed of a spatial array of imbalanced MZIs, each of which produce a linearly increasing amount of OPD to the signal, together they could produce a digitized interferogram of the input signal. This kind of configuration can easily produce a very large OPD that turns into high spectral resolution, but it suffers from a similar problem with WdM based spectrometers: inherent trade-off between spectral performance (operation bandwidth, spectral resolution) and channel count. For example, the demonstration in ref. ^83^ implements 32 MZIs for a resolution of 0.04 nm, but the bandwidth is limited to only 0.75 nm. The degree of polarization is exploited to further enhance its performance at a given channel count. A Dual-polarization SH-FTS that can process both fundamental transverse electric and transverse magnetic modes was successfully demonstrated in 2019^15^. In detail, the dual-polarized SH-FTS is composed of MZIs with different waveguide widths and arm imbalances, where each MZI can contribute two sampling OPDs, one from the TE mode and the other from the TM mode. As a consequence, the same number of MZIs can provide twice OPD compared with a single polarization operation, as shown in Fig. 10c. To further increase the spectral performance of SH-FTS at a given channel count, advanced post-processing algorithms can be explored such as compressive sensing^84^ and machine learning^85^.

The multi-interferometer configuration with a single-aperture input allows the single-shot interference capture for rapid spectral measurement, while sacrificing the Jacquinot (throughput) advantage. Since the incident light is required to be split equally into every MZI through tree-structured cascaded Y-junctions, the actual throughput for interference modulation at every OPD dramatically decreases with the interferometer number. Temporal heterodyne FTS (TH-FTS), on the other hand, is more analogous to conventional free-space based FTS, as the OPDs are modulated in time domain, and a single detector is used to capture the interferogram. The measurement time is sacrificed in order to maintain Jacquinot (throughput) advantage. Recently, two different mechanisms of achieving temporal modulation of the OPD are demonstrated. First one is similar with SH-FTS in the sense that the OPDs are also in the form of varying physical length difference, but optical switches are employed to dynamically change the physical pathway and realize the temporal modulation of OPD^86^, as shown in Fig. 10d. This approach indeed maintains the Jacquinot (throughput) advantage while produces very high spectral resolution. But the optical switches impose limit on the operation bandwidth of the spectrometer. More importantly, there will be always a leakage of signal that propagates along undesired pathway, leading to unwanted crosstalk when measuring interferograms at different OPDs, therefore introducing difficulty in the post-processing. The second approach quasi-continuously modulate the OPD by taking advantage of thermos-optic effect to change the effective index of a fixed waveguide, as shown in Fig. 10e. The challenge behind this approach mainly lies in the theoretical part dealing with various unwanted effects that only exist for waveguide structures, such as waveguide dispersion that makes different spectral components experience different OPD, fabrication variation that causes two arms of the MZI unequal, thermal induced expansions that make waveguide length varying when changing OPD etc. After successfully addressing this issues, the first experimental demonstration on silicon photonics was reported in 2018, with a bandwidth over 60 nm and a spectral resolution of only 3 nm^16^. After that, the research attention has been given to further increase its resolution. The fundamental reasons for this low resolution include short waveguide length and low power injection to the heater. Increasing waveguide length will result in large propagation loss and high electrical resistance for the heater. By using advanced waveguide design to reduce the optical loss as well as heater structures to receive higher power injection, Li et al. successfully increase the waveguide length from 3 cm in ref. ^16^ to 10 cm in ref. ^87^, and the spectral resolution is increased to sub-nanometer. Later, Li et al. combines multiple techniques to further increase the resolution to 0.16 nm, including Michelson interferometers (MI) structure to double the OPD, an optimized heater and air trenches to achieve higher thermal efficiency, a novel multiple interferometers approach is employed which combines balanced MI with N statically imbalanced MIs, thereby increasing the OPD of a single MI by factor of N + 1, as shown in Fig. 10f^17^.

#### Computational spectrometers

**Fig. 11:**
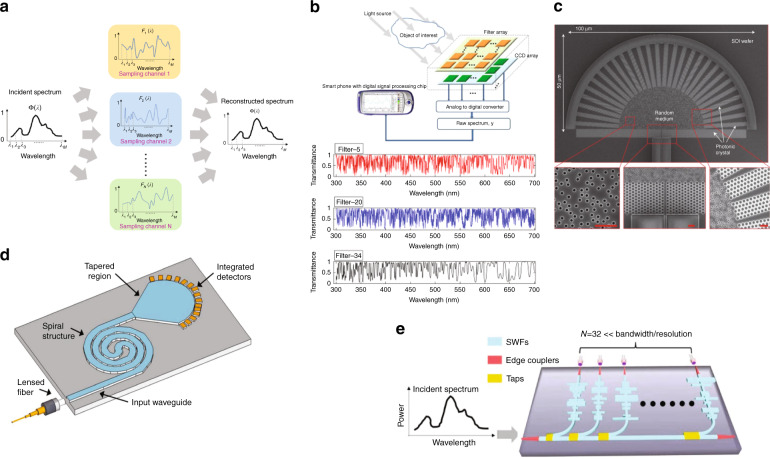
Computational spectrometers on silicon photonics platform. **a** General schematic for computational spectrometers. **b** The first demonstration of computational spectrometers^87^. **c**–**e** The recently demonstrated computational spectrometers on silicon photonics using disordered medium^13^, ultra-long multimode waveguide^96^ and stratified waveguide filters^20^, respectively. **b** Reprinted with permission from ref. ^87^. © Optica Publishing Group. **c** Reprinted by permission from Springer Nature: Nature Photonics ref. ^13^, 2013. **d** Reprinted with permission from ref. ^96^. © Optica Publishing Group. **e** Reprinted by permission from Springer Nature: Nature Communications ref. ^20^, 2021

Recently, a new scheme of WM based spectrometer named computational spectrometer has drawn extensive study, which utilizes an array of propagation channels with distinct spectral responses, each of which can be interpreted as sampling coefficients of the incident spectrum at each wavelength point, as shown in Fig. 11a. Therefore, each channel can sample the entire spectrum simultaneously. The incident spectrum can be accurately reconstructed using advanced algorithms to process the sampled results of all channels. In contrast, each channel (filter) of narrowband filters based spectrometers only samples the incident spectrum at a specific wavelength point or a small band equivalent with spectral resolution. Consequently, no advanced signal post-processing is required, but the number of channels (filters) must equal the ratio of operation bandwidth and spectral resolution. While for computational spectrometers, with proper engineering of channels’ spectral responses, the number of channels can be drastically reduced, leading to a significant improvement upon footprint and dynamic range at same bandwidth and resolution. The principle can also be mathematically explained. When a signal with a spectrum Φ(λ) propagates through a channel with spectral response F(λ) and gets detected by a photodetector, the output current is

$${{{\mathrm{I}}}} = {{{\mathrm{A}}}}{\int}_{\lambda _1}^{\lambda _M} {{{{\mathrm{F}}}}(\lambda )\Phi (\lambda ){{{\mathrm{d}}}}\lambda }$$

where A represents the link loss including the responsibility of the detector, λ_1_ and λ_M_ refer to the starting and ending wavelength of the spectrum, respectively. For signal processing purpose, the equation can be discretized as

$${{{\mathrm{I}}}} = {{{\mathrm{AF}}}}_{1xM}\Phi _{Mx1}$$

Similarly, if the signal passes through N channels with distinct F, the equation has the form of

$${{{\mathrm{I}}}}_{Nx1} = {{{\mathrm{AF}}}}_{NxM}\Phi _{Mx1}$$

For narrowband filters based spectrometer, F is a matrix with only one nonzero component at each row, thus the equation can only be solved when N = M. While for computational spectrometers, with advanced algorithms and proper F_NxM_, this equation can still be reasonably solved at the case N ≪ M, obeying the following rule:

$$minimize \, \left| {\left| {I - AF\Phi } \right|} \right|_2 \,+\, \alpha \left| {\left| {{{\Gamma }}\Phi } \right|} \right|_{{{\mathrm{2}}}} \, {{{\mathrm{subject}}}}\,{{{\mathrm{to}}}}\,0 \le \Phi \le 1$$

where α represents the coefficient of the regularization term that helps smoothen the spectrum and Γ is a matrix helping calculate the derivative of Φ, which is used for regularization purpose. At a fixed channel number (typically limited by footprint and/or SNR), the channel spectral responses will influence the quality of spectrum reconstruction significantly.

Using this concept for reconstructing signals in a specific domain is not novel. Back in 1987, researchers already proposed to design a velocimeter (a device reconstructing velocities) using random patterns^88^. In 1997 a holographic-sensor was designed using random gratings^89^. Applying this concept in signal acquisition and imaging processing has also been a well-established technique starting from early of 2000, identified as compressed sensing^90^. In the field of optical spectrometer, this concept was originally adopted only to combat with the non-ideality of narrowband filters^91–94^. In other words, these spectrometers still consist of narrowband filters, but optical span outside filters’ passbands is not zero due to fabrication imperfections, causing unwanted sampling at other wavelength span. Only till 2013, researchers theoretically verified the possibility to employ intentionally designed filters with random transmittances for improving spectrometer’s performance, as shown in Fig. 11b^95^. The main efforts since then have been put to find integrated filters or dispersive media with desirable spectral responses in order to provide good performance (large M) with only a small number of channels (small N). Generally speaking, the channel spectral responses should meet following requirements:

- Each channel’s spectral response should be linearly independent with the others to provide uncorrelated spectrum sampling.
- Each channel’s spectral response should contain adequate sharp features to provide high sampling resolution as the minimum optical distance between two distinguishable points in the spectral response determines the spectral resolution of this filter.
- The channels should be compact, low loss and be able to enjoy massive production.

It is nontrivial to get an array of filters that meet those requirements simultaneously. The first demonstration on silicon photonics was based on random media with numerous air holes distributed in the light propagation area as shown in Fig. 11c^13^. Thanks to its high level of randomness, the first two requirements were met and the spectrometer with about 30 channels can produce a spectral resolution of 0.75 nm within 25 nm bandwidth. The use of silicon photonics indeed enables massive production for such spectrometer, but the large amount of air holes introduces significant loss to the signal (as evident in Fig. 11c, most of the light scattered outside before arriving at the detectors), which makes it impossible to be used in power-sensitive applications like wearable devices for glucose and lactic acid monitoring. Also 25 nm bandwidth is inadequate for most health monitoring devices. On the other hand, researchers explore interferences instead of scatterings to create physical channels with distinct spectral responses to minimize the optical loss. An ultra-long multimode silicon waveguide with a spiral shape was developed toward computational spectrometer in 2016, plotted in Fig. 11d^96^. The interference between the modes in the waveguide as well as the intentional evanescent coupling between neighboring arms of the spiral contribute to a random spectral response at each detector. The resolution is reported to be as high as 0.01 nm but the bandwidth is relatively limited due to the very sharp speckles in the spectral responses. Moreover, the waveguide length has to be very long in order to provide rich interference patterns, which inevitably increase the device’s footprint and optical loss. Very recently, a novel computational spectrometer utilizes an array of stratified waveguide filters with distinct spectral responses was demonstrated^20,97^. The stratified waveguide filter combines the random medium theory and low-loss silicon strip waveguide to produce distinct spectral response but suppress scattering loss induced by random medium as shown in Fig. 11e. Only 32 channels were adequate to produce a bandwidth of 180 nm and a resolution of 0.45 nm. Even if the computational spectrometers exhibit distinct advantages in terms of footprint, dynamic range and measurement time, but the nature of using much less equations to solve many unknown values determines it is only good to reconstruct sparse spectra, which refer to smooth spectra or spectra with only a few nonzero components. While for dense spectra that contain rapidly changing features such a series of dips and peaks, the number of filters have to been significantly increased in order to maintain satisfying performance.

In this chapter, we review the mainstream approaches toward integrated spectrometers on silicon photonics. For summary, we present the general characteristics for different types of integrated spectrometers in Table 2.

**Table 2:** The general characteristics of different spectrometer schemes

Principle		Resolution	SNR	Dynamic range	Detection speed	Computing resource	Footprint
WdM	Dispersion	Design dependent	Low	Small	One-shot	None	Large
	Narrowband filter (spatial)	High	Low	Small	One-shot	None	Large
	Narrowband filter (temporal)	High	Moderate	Moderate	Seconds to minutes	None	Moderate
WM	Fourier transform (spatial)	Moderate	Moderate	Moderate	One-shot	High	Moderate
	Fourier transform (temporal)	High	High	Large	Seconds to hours	High	Moderate
	Reconstruction	Design dependent	Moderate	Moderate	One-shot	Moderate	Small

## Outlook

As discussed above, due to strong demand from practical applications such as biomedical sensing and industrial detection, developing integrated spectrometers with sufficient performance and massive manufacturability has never been so imperative. To date, silicon photonics is still the most promising platform due to its unique CMOS compatibility. Considering that currently demonstrated integrated spectrometers are still unsatisfiable to the market requirements, we foresee four possible directions for developing the next-generation spectrometers on silicon photonics:

## Active path scanning computational spectrometers

The rapidly growing consumer market in biomedical and chemical sensing applications have placed high spectroscopic requirements, including broad bandwidth, high resolution, and large dynamic range. However, no integrated spectrometer has so far accomplished all these merits due to respective limitations. For instance, in order to achieve broad bandwidth and high resolution, a large number of splitting channels is required for dispersive gratings, narrowband filters and SH-FTS spectrometers, which inevitably degrade the system’s dynamic range. TH-FTS doesn’t require to split the signal and can enjoy large dynamic range, but it requires a large amount of power consumption (>5 W) for sub-nm resolution, which is unacceptable for consumer electronics.

**Fig. 12:**
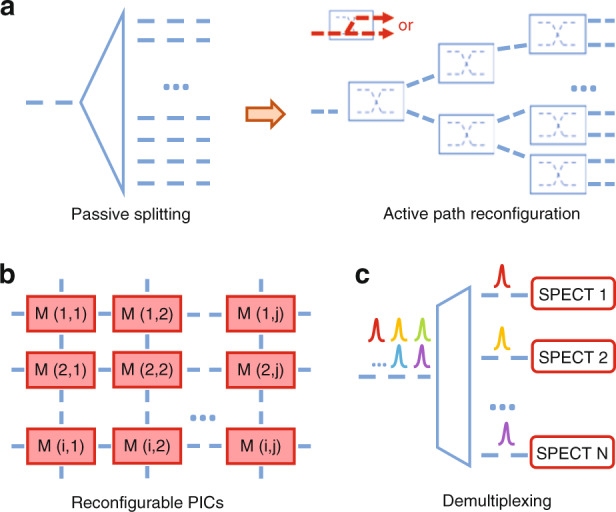
Conceptual illustrations of some promising technological paths toward the next-generation integrated spectrometers. **a** Computational spectrometers based on active path reconfiguration rather than passive power splitting. **b** Programmable spectrometers based on reconfigurable PICs. **c** Parallelism in the design of spectrometer systems

Computational spectrometers are believed promising for achieving a balanced performance with compact footprint and low power consumption, as it can significantly reduce the number of channels compared with dispersive gratings or narrowband filters based spectrometer, and do not demand power consumption compared with tunable filters spectrometers or TH-FTS. But current demonstrations still require the incident signal to be split over 30~50 sampling channels and the channel count has to scale up in order to achieve better performance, which reduces the power intensity in each channel, resulting in a worse SNR and smaller dynamic range. One potential solution is to replace the passive optical power splitting with active path scanning, that is, to actively configure different optical paths (sampling channels) for the incident signal over time, rather than splitting it over space, as shown in Fig. 12a. Consequently, a single detector is adequate to reconstruct the incident spectrum, leading to a large dynamic range for the spectrometer. Such reconfigurable switching network can be easily scaled up with exponentially increasing channel numbers to support ultra-high resolution, though at the cost of larger footprint, higher power consumption, and more complex driving circuits. Since the mainstream thermo-optic or electro-optic based optical switch technologies can achieve at least microsecond scale switching time^98,99^, the total detection time for such path-reconfigurable spectrometer can be within one second even for hundreds of detection channels.

## Spectrometer with programmability

Currently demonstrated spectrometers, irrespective of platforms and working principles, are all fixed in terms of performance, such as the center wavelength, operation bandwidth and spectral resolution. While it is highly desirable to develop a universal spectrometer that can dynamically tune its performance in order to suit requirements of different applications or working scenarios. Ideally, all of the spectrometers’ performance indicators are desired to be programmable, while in practice, it would be valuable enough to develop a spectrometer with adjustable bandwidth and resolution. This idea can be possibly realized in incorporation with programmable photonic integrated circuits (PICs)^100,101^, which is an emerging technology allowing the on-chip optical signal to be manipulated at run-time to enable various applications. As shown in Fig. 12b, a reconfigurable PIC-based spectrometer system can be established with arrayed modules which include different components such as Mach–Zehnder interferometers, ring resonators, photon detectors, and etc. By dynamically configuring the interconnections between the modules, the spectrometer architectures can be tailored for different application scenarios, e.g., the resolution can be adjusted by selecting different filter arrays. Similarly, though with sacrifices in device size and power comsuption, such designs could bring wide flexibility and versatility in practical applications.

## Parallelism in spectrometer system

Parallelism, which has always existed in the design of optical transmission system since the arise of wavelength division multiplexing (WdM) technologies^102,103^, can also be explored to enhance spectrometer’s performance. Currently, WdM is widely adopted for on-chip optical communication to increase the data volume and has been proved to be able to cover a broad bandwidth, such as C+L-band WdM^104^ and O+C-band WdM^105^. Analogically, parallelism can also be introduced into the design of spectrometer systems as shown in Fig. 12c, e.g., as a single integrated spectrometer may suffer from its limited bandwidth, by parallelly implementing a spectrometer array with different operating spectral ranges, the combined bandwidth can be significantly increased. More specifically, for applications, such as the NIR bio-sensing where various signature peaks at some specific wavelengths are to be detected, it is not realistic and also cost-worthy to build an ultra-broadband spectrometer that covers the whole spectral range with high resolution. Instead, the NIR incident signal can be firstly demultiplexed into multiple target bands (with those unwanted bands abandoned) and then detected by different spectrometers, respectively. Unlike the spatial power splitting which harms the signal intensity, the wavelength demultiplexing process does not affect the signal quality. In addition, the spectrometers targeted for different bands can be customized to match the practical performance requirements at that band. Again, such spectrometer systems will inevitably be more complicated and require more building blocks than a single spectrometer but are still highly feasible for low-cost massive production thanks to the advances in large-scale photonic integration techniques^106,107^.

## Hybrid platforms for spectrometers

Besides the design of spectrometer system itself, to realize full functionalities, it is desired to also implement high-performance light sources and photon detectors within the same chip package, which requires the introduction of other materials on the basis of silicon photonics. Currently, the mainstream integrated photodetectors are based on germanium, as it’s already a standard fabrication process in many CMOS foundries. However, Ge-on-Si photon detectors always suffer from high dark currents and low absorptivity attributed to its nature of indirect bandgap, and normally cannot cover the spectral range beyond NIR^108,109^. III-V materials, in contrast, feature direct and tunable bandgap, thus can achieve high absorption coefficients at different spectral bands. Moreover, they can also serve as gain materials for optical emitters, such as lasers^110^ or LEDs^111^. Currently, high-density cost-efficient III-V-on-Silicon integration relies on hybrid integration approaches, such as, flip-chip bonding^112^, die/wafer-to-wafer bonding^113^, and micro-transfer printing^114^. Therefore, it is predictable that hybrid integrated platforms will play an important role in the future commercialization of integrated spectrometers.

Beyond silicon photonic platforms, polymer-based 3D lithography offers new possibilities for massive producible miniaturized spectrometers. Based on the laser-induced polymerization effect, current commercial 3D nano printer can implement arbitrary three-dimensional pattern with <200 nm feature size. A variety of demonstrations of 3D-nanoprinted spectrometers have been reported based on different principles ranging from free-space optics to computational reconsctruction^115,116^. An additional design dimension could bring unique advantages for 3D nano-printed spectrometers, such as ultra-compact footprint. A wide operation bandwidth can also be expected thanks to the various ultra-broadband 3D optical coupling strategies^117^. However, at current stage, 3D nano printing only supports passive polymer structures, making external light sources and photo detectors indispensable. Therefore, by implanting the 3D nano-printed spectrometer onto the hybrid integrated III-V-on-Silicon PICs, it is possible to enjoy the advantages from both sides, representing a promising development direction.

## Conclusion

With the rapid development of Internet-of-Things and popularity of intelligent portable device, the demand for low-cost, lightweight, miniaturized spectrometers has never been so imperative. Various efforts have been made to miniaturize spectrometers, yet they somewhat lack technological readiness for commercial products. Being compatible with CMOS technology, silicon photonics is widely accepted to be one of the most promising platforms for mass-manufacturable photonic integrated circuits. Therefore, in this paper we review recent progresses in integrated spectrometers that leverage silicon photonics technology, including their market trends, application-oriented requirements with a special focus on biomedical sensing and industrial detections, and technological evolution that covers mainstream structures for silicon integrated spectrometers. Four possible research directions are foreseen for developing next-generation integrated spectrometers. We expect to see expanding integration of chip-scale spectrometers into consumer productions within the next few years, providing cost-effective and reliable services to users worldwide.
